# Hepatitis C in the Direct-Acting Antiviral Era: Immunopathogenesis, Dendritic Cells and Modern Clinical Management

**DOI:** 10.3390/biomedicines14071542

**Published:** 2026-07-09

**Authors:** Klara Kurmangaliyeva, Bakhyt Kosherova, Irina Mukatova, Aida Baibusunova, Zhanna Yeshmagambetova, Karashash Askarova, Assem Kazangapova, Raikhan Shlymova

**Affiliations:** 1 Department of Internal Diseases No. 3, Astana Medical University, Astana 010000, Kazakhstan; kkb169@mail.ru (K.K.);; 2 School of Medicine, Karaganda Medical University, Karaganda 100000, Kazakhstan; 3 Department of Internal Diseases No. 2, Astana Medical University, Astana 010000, Kazakhstan; askarova_karashash@mail.ru

**Keywords:** hepatitis C virus, hepatitis C, dendritic cells, plasmacytoid dendritic cells, HCV RNA, direct-acting antivirals, sustained virologic response, resistance-associated substitutions

## Abstract

Hepatitis C virus (HCV) infection remains a major cause of chronic liver disease worldwide, with potential progression to advanced fibrosis, cirrhosis, hepatocellular carcinoma, and extrahepatic disease. According to World Health Organization estimates, approximately 50 million people worldwide live with chronic HCV infection, and nearly 1 million new infections occur each year. In 2022, approximately 242,000 deaths were attributed to hepatitis C, mainly from cirrhosis and hepatocellular carcinoma. Chronic infection develops when antiviral immune response fails to eliminate the virus. Viral clearance requires early innate immune activation, effective antigen presentation, broad HCV-specific CD4^+^ and CD8^+^ T-cell responses and durable immune memory. Dendritic cells play a pivotal role in this process by linking innate and adaptive immunity. In chronic HCV infection, dendritic cells may be reduced in number and show impaired maturation, lower interleukin (IL)-12 production, higher IL-10 expression and weaker stimulation of HCV-specific CD4^+^ T-cell responses. This review discusses the role of dendritic cells (DCs) in HCV infection, with emphasis on DCs’ phenotype and function in acute and chronic disease. It also summarizes immune changes after direct-acting antiviral (DAA)-induced sustained virologic response (SVR), including partial recovery of innate immune responses and persistent residual immune dysregulation. When accessible and appropriately selected, modern direct-acting antiviral therapy substantially improves outcomes for patients with HCV infection.

## 1. Introduction

Hepatitis C virus (HCV) is one of the leading infectious causes of chronic liver disease. Although HCV primarily infects hepatocytes, its clinical consequences extend beyond the liver. Chronic infection may lead to progressive fibrosis, cirrhosis, hepatocellular carcinoma (HCC), liver transplantation and liver-related death [1]. According to World Health Organization (WHO) estimates, approximately 50 million people live with chronic HCV infection, with nearly 1 million new infections each year and approximately 242,000 hepatitis C-related deaths in 2022 [1]. Among patients with chronic HCV infection, the estimated risk of cirrhosis is approximately 15% to 30% within 20 years, although progression depends on age, alcohol use, metabolic disease, human immunodeficiency virus (HIV) or hepatitis B virus (HBV) coinfection, immune status and other host factors [1].

Acute hepatitis C is often mild, anicteric, or asymptomatic. Centers for Disease Control and Prevention (CDC) guidance notes that most people with HCV infection have no symptoms. Therefore, diagnosis should not rely solely on clinical presentation or reported risk factors [2].

Chronic hepatitis C is also associated with extrahepatic disease, including cryoglobulinemic vasculitis, membranoproliferative glomerulonephritis, chronic kidney disease, insulin resistance, type 2 diabetes mellitus, sicca symptoms, neuropathy, arthralgia, fatigue and B-cell lymphoproliferative disorders [3,4]. In some patients, extrahepatic manifestations precede advanced liver disease and may be the initial indication for HCV testing.

The immunopathogenesis of chronic HCV infection is shaped by both viral and host factors. Spontaneous clearance is generally associated with early innate immune activation, effective antigen presentation, and a strong, sustained, polyfunctional HCV-specific T-cell response. By contrast, chronic infection is associated with viral escape mutations, impaired interferon signaling, reduced helper T-cell support, regulatory immune signals, and progressive exhaustion of HCV-specific CD8^+^ T cells [4,5].

Several studies indicate that HCV-specific T cells do not simply disappear during chronic infection. Wieland et al. identified distinct HCV-specific CD8^+^ T-cell subsets in chronically infected patients. One subset, defined as CD127^+^PD-1^+^, had memory-like features and expressed T-cell factor 1. This subset accounted for a median of 45.7% of HCV epitope-specific CD8^+^ T-cell populations during chronic infection. Another subset, CD127-PD-1hi, showed features of terminal exhaustion [5]. Thus, chronic HCV infection contains both exhausted and memory-like antiviral T-cell populations.

Dendritic cells (DCs) are central to this immune response. Conventional DCs (cDCs), historically termed myeloid DCs (mDCs), present antigen and support T-cell differentiation. Plasmacytoid DCs (pDCs) are major producers of type I interferons during viral infection. In chronic hepatitis C, DCs may be numerically reduced and functionally impaired. Della Bella et al. reported that peripheral blood DCs from patients with chronic hepatitis C were reduced in number and showed lower IL-12 and higher IL-10 expression than DCs from healthy controls. In the same study, HCV-specific CD4^+^ T-cell proliferation was weaker and correlated with DC number and cytokine profile [6]. These findings support the concept that DC dysfunction contributes to weak antiviral immunity and viral persistence.

Plasmacytoid DCs have a dual role in HCV infection. On the one hand, they can sense HCV-infected cells and produce type I interferons. Takahashi et al. showed that HCV-infected cells trigger a strong interferon response in pDCs through a mechanism requiring active viral replication, direct cell-to-cell contact and Toll-like receptor 7 (TLR7) signaling. Supernatants from activated pDCs inhibited HCV infection in an interferon receptor-dependent manner [7]. On the other hand, patients with persistent HCV infection may have quantitative or functional pDC defects. Together, these observations highlight a central feature of HCV immunology: innate immune activation may be present but insufficiently coordinated with adaptive immunity to eliminate the virus.

Modern diagnosis should identify both previous exposure and current viremia. The CDC recommends HCV screening at least once for all adults aged 18 years and older and during each pregnancy [2]. Noninvasive fibrosis assessment is widely used before treatment. Fibrosis-4 index (FIB-4), transient elastography, platelet count, serum panels, imaging findings, and signs of portal hypertension can help identify patients with advanced fibrosis or cirrhosis. In simplified American Association for the Study of Liver Diseases/Infectious Diseases Society of America (AASLD/IDSA) algorithms, cirrhosis may be presumed when FIB-4 is greater than 3.25 or when transient elastography shows liver stiffness consistent with cirrhosis [8].

Direct-acting antiviral agents (DAAs) are now the standard of care when treatment is indicated. They target nonstructural HCV proteins essential for the viral life cycle. Compared with older interferon-based regimens, current oral regimens are shorter, better tolerated and highly effective. In most appropriately treated patients, these regimens achieve sustained virologic response (SVR), usually defined as undetectable HCV RNA 12 weeks after treatment completion (SVR12), in more than 95% of cases [1,8,9,10].

The aim of this review is to summarize the role of dendritic cells in chronic hepatitis C, as well as current diagnostic and therapeutic approaches in the era of direct-acting antiviral therapy.

## 2. Characteristics of HCV

HCV belongs to the Flaviviridae family. It is an enveloped, positive-sense, single-stranded RNA virus. Its genome is approximately 9.6 kb long and contains a single open reading frame that encodes a polyprotein of approximately 3000 amino acids. This polyprotein is subsequently cleaved by host and viral proteases into structural and nonstructural proteins [11,12].

**Figure 1:**
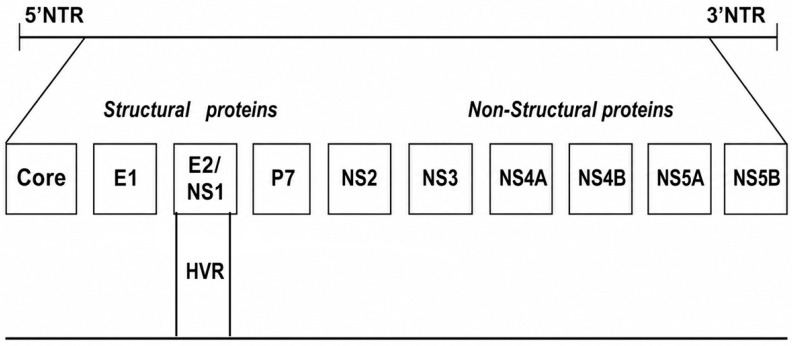
Schematic representation of the HCV RNA genome (**upper panel**) and its structural and nonstructural proteins (**lower panel**). The 5′ and 3′ untranslated regions regulate translation and RNA replication. Core forms the viral nucleocapsid. E1 and E2 mediate viral entry and contain major antibody targets, including hypervariable region 1 (HVR1). p7 is involved in assembly and release, and NS2 participates in virion assembly. NS3, with the NS4A cofactor, mediates protease and helicase functions. NS5A regulates viral RNA replication and assembly. NS5B is the HCV RNA-dependent RNA polymerase.

HCV replication occurs on modified membranes derived from the endoplasmic reticulum of infected hepatocytes. The viral genome is flanked by untranslated regions at both the 5′ and 3′ ends (Figure 1). Although these regions do not encode proteins, they are essential for translation and RNA replication. The 5′ untranslated region contains an internal ribosome entry site that enables cap-independent translation of the viral polyprotein [12].

HCV exhibits substantial genetic variability. It is currently classified into 8 genotypes and more than 90 subtypes, although the number of recognized subtypes continues to increase as sequencing data accumulate [13,14]. Genotypes differ by approximately 30% at the nucleotide level, whereas subtypes differ by about 15%. This diversity enables HCV to escape antibody pressure, form quasispecies, and persist despite antiviral immune responses [13,15].

The HCV polyprotein is processed into structural and nonstructural proteins. The structural proteins include the core protein and the envelope glycoproteins E1 and E2. The core protein forms the viral nucleocapsid, whereas E1 and E2 facilitate viral attachment and entry into host cells. The p7 protein is a small hydrophobic viroporin that supports virion assembly and release, and NS2 also contributes to viral assembly. NS3, NS4A, NS4B, NS5A, and NS5B are required for viral replication and polyprotein processing [11,15].

All HCV proteins contain epitopes that may be recognized by B cells, CD4^+^ helper T cells, and CD8^+^ cytotoxic T cells. However, HCV employs several immune-escape mechanisms, including rapid mutation, quasispecies formation, changes in neutralizing antibody targets, the tolerogenic hepatic environment, viral interference with innate immune signaling and T-cell exhaustion during persistent antigen exposure [5,15].

The envelope glycoprotein E2 is particularly important for viral entry and immune escape. Hypervariable region 1 (HVR1), located at the N-terminus of E2, consists of 27 amino acids. HVR1 is one of the most variable regions of the HCV polyprotein. Guan et al. showed that HVR1 contains functionally distinct microdomains involved in viral entry and immune evasion [16]. This supports the concept that HVR1 is both a variable antibody target and a functional region that helps HCV interact with host entry factors [16]. HVR1 also helps shield conserved neutralizing epitopes. Bankwitz et al. showed that HVR1 can modulate receptor interactions, conceal the CD81-binding site, and protect conserved neutralizing epitopes from antibody recognition [17]. These mechanisms may help explain why detectable antibody responses often fail to eliminate HCV completely.

HCV entry into hepatocytes is a multistep process involving E1/E2 and several host factors, including CD81, scavenger receptor class B type I, claudin-1, and occludin. E2 interacts with CD81 and other entry-related molecules. Regions near HVR1 can regulate exposure of the CD81-binding site, allowing the virus to balance efficient cell entry with antibody escape [17,18].

## 3. Dendritic Cell Subsets

DCs differ in origin, tissue location, surface markers, and function. Their principal role is to capture, process, and present antigens to T cells. Through this function, DCs connect innate immune recognition with adaptive immunity [19].

Human dendritic cells are commonly divided into cDCs, pDCs, and moDCs. In this review, the term cDC is used according to modern nomenclature. When older HCV studies used the term mDCs, that terminology was interpreted cautiously because older mDC definitions may include more than one modern DC population. cDCs include cDC1 and cDC2 subsets. cDC1 cells express CD141, also known as BDCA-3, and are efficient at cross-presentation and activation of CD8^+^ T cells. cDC2 cells express CD1c, also known as BDCA-1, and are important for CD4^+^ T-cell activation and cytokine-driven T-cell polarization [19,20].

Plasmacytoid DCs express CD123, CD303/BDCA-2, and CD304/BDCA-4. Their defining feature is rapid type I interferon production after viral recognition. They detect viral nucleic acids through endosomal Toll-like receptors, especially TLR7 and TLR9. For this reason, pDCs are important in early antiviral defense [19,20].

Monocyte-derived dendritic cells (moDCs) appear mainly during inflammation and are widely used in in vitro experiments. They are not identical to steady-state cDC1 or cDC2 cells. They share features with monocytes/macrophages and usually arise from circulating CD14^+^ monocytes. Their numbers may increase during infection and tissue inflammation. These cells can present antigen, produce cytokines, and regulate local immune responses [21].

Recent single-cell studies have refined the classification of human dendritic cells. They have shown that the older term “myeloid dendritic cells” includes several biologically distinct populations. For example, the CD1c^+^ compartment includes DC2 and DC3-like cells. This distinction is important when interpreting HCV studies, because older publications often used broader DC definitions. Therefore, throughout this review, mDC is used only when describing the terminology of the original studies, whereas cDC is used for modern classification [20].

## 4. Characteristics of Dendritic Cells in Acute Hepatitis C

Studying dendritic cells during acute HCV infection is challenging because most patients are asymptomatic. Consequently, clinical samples from the acute phase are limited [1,2].

Published data on circulating cDCs in acute HCV infection, historically described as mDCs, remain inconsistent. Some studies found no major change in their level, whereas others reported reduced numbers or impaired function. These differences may be related to small sample sizes, timing of blood collection, definitions of acute infection and changes in dendritic-cell nomenclature over time [22,23].

Functional studies suggest that DCs are involved early in determining whether HCV infection resolves or becomes chronic. Pelletier et al. studied patients with acute HCV infection and found that spontaneous resolution was associated with sustained DC hyperresponsiveness. In patients who subsequently cleared the virus, DCs responded more strongly to stimulation than cells from patients who progressed to chronic infection. The authors also reported that hyperresponsiveness to single-stranded RNA stimulation was sustained mainly in spontaneous resolvers [22].

Plasmacytoid dendritic cells are altered during acute HCV infection. Ulsenheimer et al. reported that circulating pDCs in acute hepatitis C had an immature phenotype, with reduced HLA-DR and CCR7 expression. These cells also produced low amounts of interferon (IFN)-alpha. In that study, median IFN-alpha production was 3.5 pg per 50,000 pDCs, indicating impaired antiviral cytokine production during early infection [23].

The association between pDC number and clinical outcome remains unclear. Some studies found reduced circulating pDCs in both patients who cleared HCV and patients who developed chronic infection, suggesting that cell number alone does not determine outcome. Sustained responsiveness to viral RNA signals appears to be more closely associated with spontaneous viral clearance [22,23].

Takahashi et al. showed that pDCs can recognize HCV-infected cells through direct cell-to-cell contact and TLR7 signaling. This response requires active viral replication and leads to type I interferon production. Interferon-containing supernatants from activated pDCs inhibited HCV infection in an interferon receptor-dependent manner [7]. Thus, pDCs can mount a strong antiviral response, although this response may remain insufficient if it is not followed by effective adaptive immunity.

## 5. Characteristics of Dendritic Cells in Chronic HCV Infection Before DAA Therapy

Dendritic cells in chronic HCV infection have been studied more extensively than those in acute HCV infection (Table 1). A few reports suggest that some DC functions are preserved in patients with chronic hepatitis C [24,25,26,27]. However, most studies show that DCs from patients with chronic HCV infection differ from those of healthy donors in both number and function [6,28,29,30,31]. These findings should be interpreted in relation to DC subset, sampling compartment, experimental method and patient characteristics.

### 5.1. Circulating and Intrahepatic Dendritic Cells

Several studies have reported reduced numbers of circulating DCs in peripheral blood. The decrease may involve cDCs/mDCs, pDCs, or both, depending on the study population and the phenotyping markers used [6,28,30,31]. Longman et al. found that circulating mDCs were lower in patients with chronic HCV than in healthy controls (0.62% versus 0.83%; *p* = 0.05), whereas the decrease in pDCs was more pronounced (0.11% versus 0.34%; *p* = 0.004) [24]. However, some functional assays in that study were preserved.

The number of circulating DCs may also be related to the activity of liver inflammation. Kunitani et al. reported an inverse correlation between circulating CD11c^+^ DC frequency and serum alanine aminotransferase activity in chronic viral hepatitis [31]. One possible explanation is compartmentalization of DCs within the liver. In patients with more active hepatitis, DCs may leave the blood and migrate into inflamed hepatic tissue. Therefore, peripheral blood studies may not fully reflect intrahepatic immunity.

Circulating and intrahepatic dendritic cells should therefore be considered related but distinct immune compartments. Peripheral blood DCs are easier to obtain and are frequently used in human HCV studies. However, they may not fully represent the hepatic immune environment, where HCV replication occurs and where DCs interact with hepatocytes, Kupffer cells, liver sinusoidal endothelial cells, stellate cells, infiltrating lymphocytes and regulatory cytokines [32,33]. Because the liver is continuously exposed to gut-derived antigens through the portal circulation, it has a naturally tolerogenic immune profile. Accordingly, hepatic DCs may show lower immunogenicity under steady-state conditions but may acquire both inflammatory and regulatory properties during chronic HCV infection [32,33].

Kunitani et al. compared circulating and intrahepatic DC subsets in chronic liver disease and showed that blood/liver DC findings were not identical. Wertheimer et al. showed that circulating mDCs and pDCs were reduced in patients with liver disease. However, this reduction may reflect altered trafficking, tissue recruitment, or systemic inflammation rather than a simple loss of DC function [33]. Nattermann et al. further suggested that interaction between HCV E2 and CD81 may contribute to abnormal DC trafficking in chronic hepatitis C [34].

Direct intrahepatic studies show that liver DCs may remain active even when peripheral blood DCs appear reduced or hyporesponsive. Lai et al. demonstrated that chronic HCV infection was associated with altered intrahepatic mDC and pDC function, indicating that local inflammatory environment affects the liver DC compartment [35]. Velazquez et al. later showed hepatic enrichment and activation of myeloid DCs during chronic HCV infection. In that study, intrahepatic mDC subsets showed features consistent with antigen presentation and immune activation, supporting the interpretation that liver DCs are not simply passive or defective during chronic infection [36]. Doyle et al. also showed that individual liver pDCs from patients with chronic HCV infection can produce IFN-alpha and multiple additional cytokines and chemokines [37]. Thus, reduced or impaired circulating pDCs do not necessarily exclude preserved or activated pDC function within the liver.

Recent single-cell studies of the human liver provide additional context. MacParland et al., Aizarani et al. and Zhao et al. showed that the human liver contains transcriptionally distinct resident and infiltrating immune-cell populations that cannot be fully inferred from peripheral blood analysis [38,39,40]. Although these studies were not limited to HCV infection, they support the broader principle that the liver has a specialized immune architecture. Peripheral blood DCs are useful for studying systemic immune changes, but they should not be considered complete surrogates for intrahepatic immunity. In chronic HCV infection, blood DCs may appear numerically reduced or functionally impaired, whereas intrahepatic DCs may be enriched, activated, polyfunctional, or regulatory. This compartmentalization may help explain why blood-based studies can underestimate the complexity of the hepatic antiviral immune response.

### 5.2. Functional Changes in Conventional and Plasmacytoid Dendritic Cells

In addition to quantitative changes, functional alterations in cDCs/mDCs have been described in chronic HCV infection. These include delayed maturation, reduced production of IL-12 and TNF-alpha, increased secretion of IL-10, reduced allostimulatory activity, and a reduced ability to support a Th1 response [6,28,29,30]. Kanto et al. reported that both myeloid and plasmacytoid blood DCs were reduced in chronic HCV infection and had an impaired ability to polarize helper T cells [28]. Della Bella et al. showed that peripheral blood DCs from patients with chronic hepatitis C had lower IL-12 and higher IL-10 expression than DCs from healthy donors. In the same study, HCV-specific CD4^+^ T-cell proliferation was less frequent and less vigorous. It correlated with DC number and IL-12 production and inversely with IL-10 expression [6]. The cytokine balance of DCs is particularly important: IL-12 supports Th1 differentiation and IFN-gamma production, whereas IL-10 limits inflammatory T-cell responses. Therefore, reduced IL-12 together with increased IL-10 may weaken HCV-specific cellular immunity and favor viral persistence.

Several studies have described a regulatory pattern of DC function. cDCs/mDCs from patients with chronic HCV infection can induce proliferation of CD4^+^CD25^+^FoxP3^+^ regulatory T cells and promote expansion of IL-10-secreting T cells in allogeneic mixed lymphocyte cultures. Dolganiuc et al. reported that mDCs from patients with chronic HCV infection induced regulatory T-cell proliferation and limited CD4^+^ T-cell proliferation through IL-2- and IL-10-dependent mechanisms [41]. Later work showed that type III interferons, IL-28 and IL-29, are increased in chronic HCV infection and can induce myeloid DC-mediated generation of FoxP3^+^ regulatory T cells [42].

For pDCs, antiviral function is linked mainly to IFN-alpha production. Many studies have reported reduced IFN-alpha secretion by circulating pDCs in chronic HCV infection [23,28,30]. Other authors, however, have found preserved IFN-alpha production [24,25]. These differences may reflect patient selection, disease activity, viral load, previous IFN-alpha/ribavirin therapy, and the type of in vitro stimulation used.

Circulating pDCs in chronic HCV infection may also have reduced allostimulatory activity and a weaker ability to activate Th1 responses [28,30]. Some studies have described increased expression of programmed death-ligand 1 (PD-L1) on these cells [30]. PD-L1 binds PD-1 on T cells and can suppress T-cell activation. This mechanism is consistent with the broader model of chronic HCV infection, in which persistent antigen exposure, IL-10-dominant regulation, and PD-1/PD-L1 signaling contribute to T-cell exhaustion and viral persistence. Like cDCs/mDCs, pDCs may also activate IL-10-secreting CD4^+^ T cells [30,41].

### 5.3. Monocyte-Derived Dendritic Cells in Chronic HCV Infection

Evidence on moDCs in chronic hepatitis C is more heterogeneous. Some studies reported impaired moDC maturation, increased PD-L1 expression, reduced production of proinflammatory cytokines and IFN-alpha, increased IL-10 secretion, reduced allostimulatory activity and a weaker ability to induce Th1 responses [29,30,43,44]. Bain et al. reported impaired allostimulatory function of moDCs in chronic hepatitis C [43]. MacDonald et al. later showed that moDC function may appear impaired when tested at physiologic DC numbers, suggesting that experimental conditions can strongly influence the results [44].

At the same time, several studies found preserved moDC function. Piccioli et al. reported that immature moDCs from patients with chronic HCV retained allostimulatory activity, maturation capacity, and TNF-alpha production after lipopolysaccharide (LPS) stimulation [25]. Barnes et al. found no significant differences between moDCs from patients with chronic HCV and healthy donors in surface-marker expression, IL-10 and IL-12p70 production, allostimulatory activity, or the ability of mature moDCs to induce expansion of antigen-specific CD8^+^ T cells [26].

Longman et al. also reported preserved maturation and functional activity of moDCs in chronic HCV infection. In their study, mature DCs from 13 of 13 patients with chronic HCV expressed typical maturation markers and were able to prime allogeneic T cells and stimulate influenza-specific memory T cells [27]. Canaday et al. similarly demonstrated preserved MHC-II antigen processing and presentation in chronic HCV infection [45]. Overall, patients with chronic HCV infection are not generally immunodeficient; rather, they fail to mount an effective immune response against HCV itself.

### 5.4. Factors Contributing to Heterogeneity Among Studies

The heterogeneity in the DC literature likely reflects both biological and methodological factors. Older studies frequently used broad mDC gates that may include several modern populations, including cDC1, cDC2, and DC3-like cells. Functional assays also differed substantially: some studies measured allogeneic mixed lymphocyte reactions, whereas others assessed HCV-specific T-cell responses, cytokine secretion after TLR stimulation, antigen processing, or maturation markers. Preserved allostimulatory or antigen-presenting capacity does not exclude defective HCV-specific Th1 priming or altered cytokine polarization [6,24,25,26,27,28,30,45].

The in vitro stimulus used is another important source of variability. CpG, poly(I:C), LPS, recombinant HCV proteins, cell-free virus, and HCV-infected cells activate different sensing pathways. This issue is especially relevant for pDCs because HCV recognition may require direct contact with infected hepatocytes, active viral replication, and TLR7 signaling—conditions that are not reproduced by simple exposure to soluble ligands [7].

Patient characteristics may also contribute to inconsistent findings. Chronic HCV infection varies by viral load, genotype, alanine aminotransferase (ALT) activity, fibrosis stage, cirrhosis status, metabolic disease, alcohol exposure, coinfections, and previous treatment history. Circulating CD11c^+^ DC frequency has been reported to correlate inversely with ALT activity, suggesting that active hepatic inflammation may be associated with redistribution of DCs from blood to liver [31]. Therefore, cohorts with active hepatitis or advanced liver disease may show reduced circulating DCs even when intrahepatic DCs are recruited or activated.

Finally, many older studies were performed during the interferon/ribavirin era and included mixed populations of treatment-naive patients, nonresponders, and previously treated patients. Interferon-based therapy can alter DC maturation, cytokine secretion, and T-cell stimulatory capacity. In addition, peripheral blood studies do not necessarily represent the hepatic immune environment because DCs may redistribute among blood, lymphoid tissue and inflamed liver tissue [34,37]. These factors should be considered when interpreting whether DC function is truly impaired or instead altered in a compartment- and assay-dependent manner.

**Table 1:** Characteristics of dendritic cells in chronic HCV infection.

DC Subset	Findings	Assay Type	References
cDCs (mDCs)	Preserved functional capacity	Peripheral blood ex vivo study	[24]
	↓ circulating cell number	Peripheral blood ex vivo studies	[6,24,28,30,31]
	Impaired maturation	Peripheral blood ex vivo study	[30]
	↓ IL-12 and/or TNF-alpha production; ↑ IL-10 production	Peripheral blood ex vivo studies	[6,28,30]
	↓ allostimulatory activity and/or ↓ Th1-stimulatory activity	Peripheral blood ex vivo studies	[6,28,30]
	↑ PD-L1 expression and/or induction of IL-10-producing T cells	Ex vivo-purified DC subset studies and mixed lymphocyte cultures	[30,41]
	↑ stimulation of CD4^+^CD25^+^FoxP3^+^ regulatory T-cell proliferation	Myeloid DC/regulatory T-cell experiments	[41,42]
	Circulating CD11c^+^ DC number inversely correlated with ALT	Peripheral blood and intrahepatic comparison in chronic liver disease	[31]
pDCs	Preserved IFN-alpha production or preserved functional capacity in selected studies	Peripheral blood pDC stimulation assays	[24,25]
	↓ circulating cell number	Peripheral blood ex vivo studies	[6,23,24,28,30]
	↓ IFN-alpha production	Peripheral blood pDC stimulation assays	[23,28,30]
	↓ allostimulatory activity and/or ↓ Th1-stimulatory activity	Peripheral blood ex vivo studies	[28,30]
	↑ PD-L1 expression and/or altered regulatory phenotype	Ex vivo-purified pDC subset studies	[30]
	pDC number directly correlated with IFN-alpha production	Intrahepatic study	[37]
moDCs	Maturation and cytokine production preserved in selected studies	Monocyte-derived DC experiments	[25,26,27]
	Impaired maturation or impaired function under selected experimental conditions	Monocyte-derived DC experiments	[29,43,44]
	↓ allostimulatory activity	Monocyte-derived DC experiments	[43,44]
	↓ IL-12 and/or altered immunostimulatory cytokine function	Monocyte-derived DC experiments	[29,43,44]
	↑ IL-10 or regulatory T-cell-promoting effects	Monocyte-derived DC experiments	[41,42]
	Preserved allostimulatory activity in selected studies	Monocyte-derived DC experiments	[25,26,27]
	Preserved antigen-specific T-cell stimulation	Monocyte-derived DC experiments	[26,27]

Note: ↑, increase; ↓, decrease. cDC, conventional dendritic cell; mDC, myeloid dendritic cell (used in older studies); pDC, plasmacytoid dendritic cell; moDC, monocyte-derived dendritic cell; ALT, alanine aminotransferase; IFN, interferon; IL, interleukin; PD-L1, programmed death-ligand 1; Th1, T helper type 1.

## 6. Diagnostic Assessment Before Antiviral Therapy

Modern HCV diagnosis should establish whether the patient has previous exposure, current active infection, and clinically relevant liver disease. The initial test is an anti-HCV antibody assay. A reactive antibody test indicates previous exposure but does not distinguish resolved infection from current viremia. Therefore, a reactive antibody result should be followed by nucleic acid testing for HCV RNA. Detectable HCV RNA confirms current infection and should prompt counseling, treatment evaluation, and selection of an appropriate DAA regimen [2,8].

The CDC recommends universal HCV screening at least once for all adults aged 18 years and older and for all pregnant persons during each pregnancy. People with ongoing risk factors should be tested periodically. HCV RNA testing after a reactive antibody result is preferred because it reduces loss to follow-up and shortens the pathway from screening to diagnosis [2].

Pretreatment laboratory assessment commonly includes a complete blood count, hepatic function panel, estimated glomerular filtration rate, quantitative HCV RNA, HIV antigen/antibody testing, hepatitis B surface antigen testing and medication reconciliation to identify drug–drug interactions [8,9]. HBV status is clinically important because HBV reactivation has been reported during DAA therapy in HBV/HCV-coinfected patients [10]. Therefore, patients should be tested for HBV infection before starting DAAs and monitored when indicated [10,46]. Patients with decompensated cirrhosis, hepatocellular carcinoma, pregnancy, prior DAA failure, liver transplantation, or complex drug interactions require individualized management.

## 7. Current Antiviral Therapy for Hepatitis C

**Figure 2:**
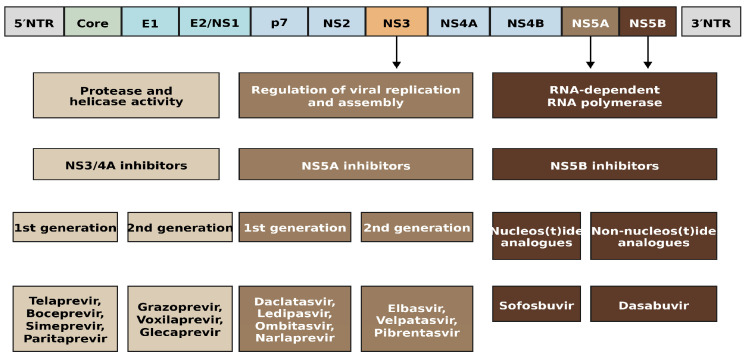
Direct-acting antiviral drugs used to treat hepatitis C virus infection.

DAAs target specific nonstructural HCV proteins that are essential for viral replication, polyprotein processing and virion assembly. There are three main DAA classes, each directed against a different viral target. Pharmacologic class can usually be recognized by the suffix of the international nonproprietary name: “-previr” indicates an NS3/4A protease inhibitor, “-buvir” indicates an NS5B RNA-dependent RNA polymerase inhibitor, and “-asvir” indicates an NS5A inhibitor (Figure 2).

Modern antiviral therapy is based on combinations of DAAs from different classes. This approach increases antiviral potency, reduces the risk of resistance, and allows short, interferon-free regimens. In current guidelines, pangenotypic regimens are preferred for many patients because they simplify treatment selection and expand access to care [8]. WHO also recommends pangenotypic DAA therapy for adults, adolescents and children aged 3 years and older with chronic HCV infection [47].

### 7.1. Direct-Acting Antiviral Agents Targeting the HCV NS3/4A Protein

The first approved NS3/4A protease inhibitors were telaprevir and boceprevir. Approved in 2011 for chronic HCV genotype 1 infection, they were used only in combination with pegylated IFN-alpha and ribavirin. In the ADVANCE trial, telaprevir-based therapy achieved SVR in up to 75% of patients, compared with 44% in the control group. In SPRINT-2, boceprevir-based therapy achieved SVR rates of approximately 67–68%, compared with 40% with peginterferon/ribavirin alone [48,49].

These drugs are no longer used in routine HCV treatment. Their use was limited by frequent adverse events, drug interactions, high pill burden and a low-to-moderate genetic barrier to resistance. In real-world cohorts, telaprevir/boceprevir-based therapy was associated with anemia, rash, treatment modification, and discontinuation, especially in patients with advanced liver disease [50].

The genetic barrier to resistance is an important characteristic of DAA regimens. It reflects the number of amino acid substitutions required in the viral target protein for clinically meaningful resistance to develop. Drugs with a low resistance barrier may lose activity after one or two substitutions, whereas drugs with a high barrier usually require several substitutions in the same functional region. Telaprevir and boceprevir had a low-to-moderate resistance barrier [51,52].

Later NS3/4A protease inhibitors include grazoprevir, voxilaprevir, and glecaprevir. Glecaprevir- and voxilaprevir-containing regimens have broad pangenotypic activity, whereas grazoprevir, used with elbasvir, is primarily used for selected patients with genotype 1 or 4 infection and should not be considered fully pangenotypic [8,9]. In studies of elbasvir/grazoprevir, SVR rates were generally above 90% in appropriate genotype 1 and 4 populations, with good tolerability [53]. Glecaprevir/pibrentasvir has demonstrated high efficacy across genotypes 1 to 6. In the ENDURANCE studies, SVR reached 99.1% after 8 weeks and 99.7% after 12 weeks in genotype 1 infection; in genotype 3 infection, SVR12 was approximately 95% [54].

A clinically important limitation of all NS3/4A protease inhibitor-containing regimens is their liver safety profile in patients with decompensated cirrhosis. Regimens containing simeprevir, paritaprevir, grazoprevir, glecaprevir, or voxilaprevir should not be used in patients with Child–Pugh class B or C cirrhosis or in those with a history of hepatic decompensation [8,9,55].

### 7.2. Direct-Acting Antiviral Agents Targeting the HCV NS5A Protein

First-generation NS5A inhibitors include daclatasvir, ledipasvir, and ombitasvir [56,57]. In early interferon-based regimens, daclatasvir combined with ribavirin and peg-IFN-alpha improved SVR in genotype 1 infection. More importantly, daclatasvir combined with sofosbuvir produced high SVR rates in several genotypes. In a landmark study, SVR12 reached 98% in genotype 1, 92% in genotype 2, and 89% in genotype 3 infection [58].

Ledipasvir was developed as a fixed-dose combination with sofosbuvir. In the ION-1 trial, ledipasvir/sofosbuvir achieved SVR12 in approximately 97–99% of treatment-naive patients with genotype 1 infection [59]. This regimen was initially used for genotype 1 infection and later also for genotypes 4, 5, and 6. Ombitasvir is used with paritaprevir/ritonavir and dasabuvir. In phase 3 studies, this combination achieved SVR12 rates above 95% in genotype 1 infection, with or without ribavirin, depending on subtype and cirrhosis status [60].

Second-generation NS5A inhibitors include elbasvir, velpatasvir, and pibrentasvir. Elbasvir is used with grazoprevir and is mainly relevant to genotype 1 and 4 infection. Velpatasvir, combined with sofosbuvir, provides pangenotypic activity. In ASTRAL-1, sofosbuvir/velpatasvir for 12 weeks achieved SVR12 in 99% of patients with HCV genotypes 1, 2, 4, 5, and 6 [61]. Pibrentasvir, combined with glecaprevir, also has pangenotypic activity. In appropriately selected patients with genotypes 1 to 6, glecaprevir/pibrentasvir achieves SVR12 in more than 95% of cases [8,54].

New NS5A-containing regimens continue to be studied. The combination of ruzasvir, an investigational NS5A inhibitor, with bemnifosbuvir, a nucleotide polymerase inhibitor, has been evaluated in phase 2 studies [62]. This regimen remains investigational and should not be presented as standard guideline-based treatment.

### 7.3. Direct-Acting Antiviral Agents Targeting the HCV NS5B Protein

There are two main groups of NS5B inhibitors. The first group comprises nucleos(t)ide analogs [63]. At present, the main clinically used drug in this class is sofosbuvir. Sofosbuvir is a prodrug that undergoes intracellular metabolism to an active triphosphate form. It mimics a natural nucleotide substrate, is incorporated into the growing viral RNA chain, and causes chain termination. Because the NS5B active site is highly conserved across HCV genotypes, nucleotide polymerase inhibitors are pangenotypic, highly effective, and characterized by a high genetic barrier to resistance.

Sofosbuvir was approved for the treatment of chronic hepatitis C in 2013. It was studied with ribavirin and peg-IFN-alpha and later in combination with other DAAs. In the NEUTRINO study, sofosbuvir combined with peginterferon/ribavirin achieved SVR12 in 90% of patients with HCV genotypes 1, 4, 5, or 6 [64]. Sofosbuvir was also the first DAA widely used as the backbone of interferon-free treatment regimens [65].

The second group consists of non-nucleoside NS5B inhibitors. Compared with nucleos(t)ide inhibitors, these agents generally have narrower genotype coverage, lower antiviral potency, and a lower resistance barrier because they bind less conserved polymerase regions. The best-known representative is dasabuvir, which was used to treat chronic HCV genotype 1 infection as part of the paritaprevir/ritonavir/ombitasvir plus dasabuvir regimen [60].

## 8. HCV Treatment Regimens Containing Direct-Acting Antiviral Agents

Current HCV treatment regimens generally combine at least two DAAs from different classes. In appropriately selected patients, DAA-based regimens achieve SVR12 in more than 95% of cases [1,8,9]. The usual duration of therapy is 8 to 12 weeks for most patients, depending on regimen, cirrhosis status, previous treatment history, genotype when relevant, and other clinical factors. WHO recommends simplified care pathways and pangenotypic DAA regimens to expand access to HCV treatment [47].

Regimens approved by major regulatory agencies or used in international and regional practice include sofosbuvir/velpatasvir, glecaprevir/pibrentasvir, elbasvir/grazoprevir, paritaprevir/ritonavir/ombitasvir plus dasabuvir, and sofosbuvir/velpatasvir/voxilaprevir. These regimens should not be considered interchangeable. Sofosbuvir/velpatasvir and glecaprevir/pibrentasvir are current pangenotypic options for many first-line patients. Sofosbuvir/velpatasvir/voxilaprevir is mainly a pangenotypic retreatment option for selected patients with previous DAA failure [8,9].

In patients with decompensated cirrhosis, corresponding to Child–Pugh class B or C, regimens containing NS3/4A protease inhibitors should not be prescribed. This includes simeprevir, paritaprevir, grazoprevir, glecaprevir, and voxilaprevir [8,9,55]. In such patients, sofosbuvir-based regimens with an NS5A inhibitor are generally preferred, and ribavirin may be added when tolerated and clinically appropriate.

Before prescribing DAAs, clinicians should evaluate potential drug–drug interactions with medications used for comorbid conditions. Clinically important interactions may occur with statins, antidepressants, anticonvulsants, antiarrhythmic agents, immunosuppressants, antiretroviral drugs and acid-reducing agents [66].

Current AASLD/IDSA simplified algorithms recommend two first-line options for eligible treatment-naive adults without cirrhosis: glecaprevir/pibrentasvir for 8 weeks or sofosbuvir/velpatasvir for 12 weeks (Table 2) [67,68,69]. For treatment-naive adults with compensated cirrhosis, glecaprevir/pibrentasvir is recommended for genotypes 1–6, whereas sofosbuvir/velpatasvir is recommended for genotypes 1, 2, 4, 5, and 6 because genotype 3 requires baseline NS5A RAS testing [68].

In patients with decompensated cirrhosis, protease inhibitor-containing regimens are not recommended because of liver safety concerns. For patients with decompensated cirrhosis and genotype 1–6 infection, sofosbuvir/velpatasvir plus ribavirin for 12 weeks is recommended when ribavirin can be used. If ribavirin is unavailable or not tolerated, sofosbuvir/velpatasvir for 24 weeks is recommended [55].

## 9. HCV Resistance-Associated Substitutions and Direct-Acting Antiviral Agents

HCV replicates rapidly, and its RNA-dependent RNA polymerase lacks proofreading activity. As a result, viral variants are generated continuously during replication [70]. Most substitutions have no clinical effect, but some may produce resistance-associated substitutions (RASs) that reduce susceptibility to one or more DAAs. The clinical relevance of a RAS depends on the drug class, HCV genotype or subtype, the number of substitutions present and the regimen used [71].

Clinically relevant resistance to nucleos(t)ide NS5B inhibitors is uncommon because the catalytic site of NS5B is highly conserved. The classic sofosbuvir-associated substitution is S282T, in which serine is replaced by threonine at position 282. This substitution reduces susceptibility to sofosbuvir but also markedly reduces viral fitness. For this reason, S282T is rare after sofosbuvir failure and disappears after drug exposure is removed [51,71].

Long-term persistence of NS5A RASs has direct implications for retreatment. Pawlotsky noted that NS3/4A-resistant variants usually disappear from peripheral blood within weeks to months, whereas NS5A inhibitor-resistant variants may persist for years [51]. Wyles et al. confirmed this pattern after failure of ledipasvir-containing therapy, showing that most treatment-emergent NS5A substitutions remained detectable for more than 96 weeks after treatment failure [72].

Clinically relevant NS5A substitutions depend on genotype and regimen. In genotype 1a, substitutions at M28, Q30, L31, and Y93 are important for ledipasvir, elbasvir, and other NS5A inhibitors. In genotype 3, Y93H and A30K are especially important because they may reduce susceptibility to velpatasvir or pibrentasvir in selected clinical settings [71,73].

In DAA-naive patients, baseline RASs that clearly alter treatment choice are relatively uncommon. Therefore, routine resistance testing is not required before most first-line pangenotypic regimens. AASLD/IDSA guidance recommends RAS testing mainly when it will affect regimen selection, especially before retreatment or in selected patients with genotype 3 infection and cirrhosis when sofosbuvir/velpatasvir is being considered [8,68,71]. If Y93H is present, ribavirin should be added or another recommended regimen should be selected [68].

For many patients with prior DAA failure, especially after an NS5A-containing regimen, sofosbuvir/velpatasvir/voxilaprevir for 12 weeks is the main pangenotypic retreatment option when decompensated cirrhosis is absent [8,9]. In the POLARIS studies, this triple-class regimen was highly effective in DAA-experienced patients [74]. Real-world data also support the high effectiveness of sofosbuvir/velpatasvir/voxilaprevir after DAA failure [75]. Retreatment is more complex in patients with genotype 3 infection, cirrhosis, prior sofosbuvir/velpatasvir failure, or multiple DAA failures. For patients with multiple DAA failures, including failure of sofosbuvir/velpatasvir/voxilaprevir, recommended options may include glecaprevir/pibrentasvir plus sofosbuvir and ribavirin for 16 weeks, with extension to 24 weeks in difficult cases. Alternative includes sofosbuvir/velpatasvir/voxilaprevir plus ribavirin for 24 weeks in selected patients [76]. Triple-therapy failure is rare but clinically important, and RAS-guided salvage therapy can be successful in selected cases [77]. Therefore, previous NS5A exposure should guide retreatment selection even years after failure.

## 10. Monitoring the Effectiveness of Antiviral Therapy

Patients should be informed that successful antiviral therapy does not protect against reinfection. Reinfection is possible after new exposure to HCV. Therefore, patients with ongoing risk factors should undergo periodic HCV RNA testing after SVR12. AASLD/IDSA recommends annual HCV RNA testing for patients with ongoing reinfection risk because anti-HCV antibodies usually remain positive and cannot distinguish past cure from new active infection [8].

Patients without cirrhosis who achieve SVR12 and have no ongoing risk of reinfection usually do not require liver-specific follow-up. In patients with absent to moderate baseline fibrosis, corresponding to METAVIR F0-F2, further HCV-specific follow-up is generally unnecessary if HCV RNA remains undetectable and liver tests are normal [8,9,78].

Patients with advanced fibrosis or cirrhosis require long-term surveillance even after SVR12. The 2024 European Association for the Study of the Liver position paper on follow-up after HCV cure states that patients with advanced fibrosis or cirrhosis who achieve SVR should continue surveillance because the risk is reduced but not eliminated [78]. AASLD HCV guidance also supports surveillance in adults with cirrhosis using liver ultrasound, with or without alpha-fetoprotein, every 6 months [79].

Thus, post-treatment assessment should not be limited to HCV RNA negativity. It should also consider fibrosis stage, cirrhosis status, reinfection risk, liver enzyme activity, and the need for ongoing HCC surveillance.

## 11. Immune Responses After DAA Therapy

Viral clearance reduces the persistent antigenic stimulus generated by HCV RNA, viral proteins and infected hepatocytes. Consequently, SVR is usually followed by improvement in liver inflammation, reduction in interferon-inducible chemokines, and partial normalization of innate and adaptive immune activation [80,81,82,83,84].

For DCs, post-treatment data remain more limited than pretreatment evidence. Nevertheless, these data are central to understanding HCV immunology in the DAA era. During the transition from interferon-based therapy to DAA-containing therapy, Sacchi et al. showed that dendritic-cell activation was associated with sustained virologic response during telaprevir-based treatment [84]. However, telaprevir was used with pegylated interferon and ribavirin, so these results cannot be interpreted as the direct immunologic effect of interferon-free DAA therapy alone.

More recent interferon-free DAA studies suggest that recovery of the circulating DC compartment after SVR is partial and subset-dependent. Zuccalà et al. performed a longitudinal analysis of HCV-monoinfected and HIV/HCV-coinfected patients before DAA therapy and at SVR12 [85]. They evaluated multiple immune-cell populations, including pDCs and mDCs. Their data showed that DAA therapy did not produce uniform changes across all DC subpopulations. In particular, mDC deficits observed in HIV/HCV-coinfected patients appeared to improve after SVR, whereas pDC changes were less consistent and inflammatory/tissue-remodeling markers such as sCD163 and MMP-2 could remain abnormal. Thus, DAA therapy may improve some circulating DC parameters and the inflammatory environment in which DCs function. Although current evidence is insufficient to conclude that DC functions such as IL-12 production, pDC IFN-alpha release, antigen processing, and HCV-specific T-cell priming are fully restored after SVR [85].

A recent study also suggested that DAA therapy can modify CD200R^+^ DC frequencies, further supporting the interpretation that DC recovery is measurable but incomplete and context dependent [86].

During active chronic infection, HCV continuously stimulates innate immune pathways, including interferon-stimulated genes, TLR-dependent sensing, monocyte/macrophage activation, and regulatory cytokine production. After DAA-mediated viral clearance, this stimulus rapidly decreases. As a result, interferon-inducible chemokines such as CXCL10/IP-10 usually decline, and markers of monocyte/macrophage activation, such as soluble CD163 and soluble CD14, may improve [82,83,85]. Mascia et al. showed that CXCL10, sCD163, and sCD14 were increased before treatment and that CXCL10 and sCD163 decreased in patients who achieved SVR, whereas they remained elevated in nonresponders [83]. These findings suggest that declining viremia is closely linked to reduced innate immune activation.

Other innate and adaptive immune compartments also show partial restoration after successful DAA therapy. NK-cell responsiveness to IFN-alpha improves as HCV viremia declines [87]. Reduced lymphocyte activation and partial reversal of exhausted T-cell phenotypes have also been described [81]. Szereday et al. found that DAA therapy was associated with reduced expression of inhibitory immune-checkpoint markers, including TIM-3, PD-L1, and Galectin-9 [88]. Because chronic innate activation and excessive checkpoint signaling may impair T-cell effector function and antigen presentation, these changes may improve DC-T-cell interaction.

Despite these improvements, residual immune dysregulation after SVR is well documented. Hengst et al. showed that successful DAA-induced HCV clearance reduced many elevated cytokines and chemokines but did not restore the entire soluble immune mediator profile to the level observed in non-HCV controls [82]. Vranjkovic et al. reported persistent abnormalities in circulating CD8^+^ T-cell subsets after DAA therapy in patients with advanced liver fibrosis, suggesting that advanced liver disease may maintain a systemic immune-activating environment even after HCV RNA becomes undetectable [89]. This finding is clinically relevant because patients with advanced liver disease remain at risk of liver-related complications after SVR.

Residual immune changes are also observed in adaptive immunity. DAA therapy can improve HCV-specific CD8^+^ T-cell function, proliferation, and cytokine production, especially when functional memory-like T-cell populations are still present before treatment [5,80]. However, chronic antigen exposure may leave persistent exhaustion-associated and epigenetic changes. Aregay et al. reported that exhausted HCV-specific CD8^+^ T cells may remain functionally and metabolically impaired despite viral clearance [90]. Yates et al. showed that exhausted HCV-specific CD8^+^ T cells retain an exhaustion-associated chromatin landscape after curative therapy. In particular, super-enhancer regions near exhaustion-related genes, including *TOX* and *HIF1A*, remained epigenetically scarred, suggesting that chronic antigen stimulation may induce stable immune programs that persist even after antigen exposure [91]. Kim et al. extended this concept to regulatory T cells by showing that inflammatory Treg features may also be retained after successful DAA treatment [92]. These findings are relevant to dendritic-cell biology because DCs maintain and reactivate antiviral T-cell responses. If the T-cell compartment remains partially exhausted after SVR, complete functional recovery of the DC-T-cell axis may not occur.

## 12. Review Limitations and Future Directions

This review has several limitations. It is a narrative rather than a systematic review, and literature selection was not based on a registered protocol. Many DC studies used small cohorts, heterogeneous patient populations, different fibrosis stages, varied treatment histories, and nonuniform definitions of DC subsets. Peripheral blood evidence may not accurately represent the intrahepatic immune environment, and post-DAA studies of DC function remain fewer than pretreatment immunopathogenesis studies.

This review also has several strengths. It integrates virologic, immunologic, diagnostic, therapeutic, resistance-related, and post-SVR aspects of HCV in the DAA era. It distinguishes modern DC nomenclature from historical mDC terminology, separates peripheral blood, intrahepatic, and moDC evidence, and places early DAA development within the context of currently recommended regimens and updated follow-up guidance.

Future studies should use standardized single-cell and multiparametric methods to compare circulating and intrahepatic DC subsets before treatment, during therapy, and after SVR12. Priorities include determining whether residual DC dysregulation contributes to persistent liver inflammation, reinfection susceptibility, impaired vaccine responsiveness, or hepatocellular carcinoma risk. Further work is also needed to optimize resistance-informed retreatment and long-term surveillance for patients with advanced fibrosis or cirrhosis after SVR.

## 13. Conclusions

The last decade has marked a major shift in clinical pharmacology and hepatology. Highly effective oral DAAs have replaced prolonged IFN-based regimens and made it possible to cure chronic hepatitis C in more than 95% of appropriately treated patients. However, DAA-induced SVR represents virologic cure rather than complete immune normalization.

Chronic HCV infection is now curable, but elimination still depends on early diagnosis, treatment access, careful management of special patient groups, prevention of reinfection, and continued follow-up of patients with advanced liver disease. HCV remains important because it illustrates how viral persistence can develop despite preserved immune competence. Dendritic-cell dysfunction, regulatory immune signaling, and T-cell exhaustion may help explain why the host immune response fails to eradicate HCV. At the same time, DAA-induced cure provides an opportunity to study partial immune restoration after removal of chronic viral antigen exposure.

## Figures and Tables

**Table 2:** Recommended first-line DAA regimens for treatment-naive adults with chronic HCV infection.

Patient Group	Recommended Regimen	Duration	Genotype Coverage
Treatment-naive adults without cirrhosis	Glecaprevir/pibrentasvir	8 weeks	Genotypes 1–6
Treatment-naive adults without cirrhosis	Sofosbuvir/velpatasvir	12 weeks	Genotypes 1–6
Treatment-naive adults with compensated cirrhosis, Child–Pugh A	Glecaprevir/pibrentasvir	8 weeks	Genotypes 1–6
Treatment-naive adults with compensated cirrhosis, Child–Pugh A	Sofosbuvir/velpatasvir	12 weeks	Genotypes 1, 2, 4, 5, and 6; genotype 3 after NS5A substitution testing
Decompensated cirrhosis, Child–Pugh B or C; ribavirin eligible	Sofosbuvir/velpatasvir plus ribavirin	12 weeks	Genotypes 1–6
Decompensated cirrhosis, Child–Pugh B or C; ribavirin ineligible	Sofosbuvir/velpatasvir	24 weeks	Genotypes 1–6

## Data Availability

No new data were created or analyzed in this study.

## Abbreviations

AASLD/IDSA	American Association for the Study of Liver Diseases/Infectious Diseases Society of America
ALT	Alanine aminotransferase
cDC	Conventional dendritic cell
DAA	Direct-acting antiviral
DC	Dendritic cell
EASL	European Association for the Study of the Liver
HBsAg	Hepatitis B surface antigen
HBV	Hepatitis B virus
HCC	Hepatocellular carcinoma
HCV	Hepatitis C virus
HIV	human immunodeficiency virus
HVR1	Hypervariable region 1
IFN	Interferon
IL	Interleukin
mDC	Myeloid dendritic cell; historical term used in older studies and interpreted here within modern cDC nomenclature when appropriate
moDC	Monocyte-derived dendritic cell
NK	Natural killer
NS	Nonstructural protein
pDC	Plasmacytoid dendritic cell
PD-1	Programmed cell death protein 1
PD-L1	Programmed death-ligand 1
RAS	Resistance-associated substitution
sCD14	Soluble CD14
sCD163	Soluble CD163
SVR	Sustained virologic response
SVR12	Sustained virologic response 12 weeks after treatment completion
TLR	Toll-like receptor
Treg	Regulatory T cell
CXCL10/IP-10	C-X-C motif chemokine ligand 10/interferon gamma-induced protein 10
WHO	World Health Organization
